# Emergent Redo Transcatheter Aortic Valve Implantation in a Nonagenarian Patient with Multiple Organ Failure

**DOI:** 10.1016/j.cjco.2025.08.014

**Published:** 2025-09-01

**Authors:** Sarah Mauler-Wittwer, Bernado Pinto, Andres Hagermann, Georgios Giannakopoulos, Stephane Noble

**Affiliations:** a Department of Medicine, Cardiology Division, Structural Heart Unit, University Hospitals of Geneva, Geneva, Switzerland; b Department of Acute Medicine, Anesthesiology Division, University Hospitals of Geneva, Geneva, Switzerland

**Keywords:** multiple organ failure, Redo-TAVI, elderly

A 97-year-old woman was admitted for acute heart failure and pneumonia. Seven years earlier, she had undergone transcatheter aortic valve implantation (TAVI) with 29-mm Evolut-Pro transcatheter heart valves (THVs) (Medtronic, MN) in series because of high gradient symptomatic severe aortic stenosis with bicuspid anatomy (calcium score 3800 HU; Fig. 1A). The first 29-mm Evolut-Pro THV was correctly positioned until the final deployment, at which point it popped up, probably due to a lack of sufficient tension release in the system (Fig. 1, B and C; Video-1A , view video online). Transthoracic echocardiography and invasive assessment (Video 1 B , view video online) showed a stable valve with moderate paravalvular aortic regurgitation (AR) and a mean gradient of 17 mm Hg. Considering the large sinuses of Valsalva (30 x 33 x 37 mm), we implanted a second THV without pulling away the first one (Video 1, C and D , view video online). Crossing the first THV was challenging. Finally, the wire was advanced using a transseptal approach; it was snared and exteriorized in the groin through the right femoral artery. The second THV was successfully deployed. The evolution was favourable (mean gradient: 5 mm Hg, with no AR), with resolution of dyspnea up to the 6-year follow-up.

**Figure 1:**
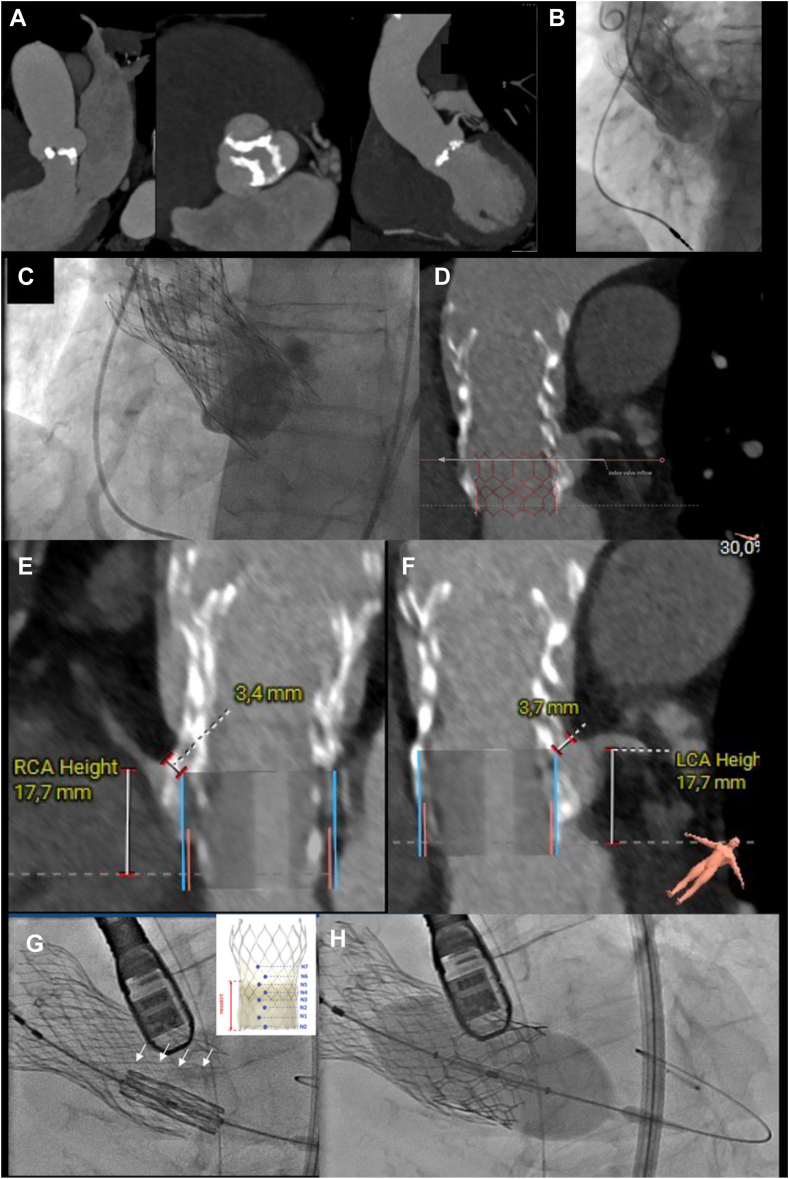
(**A**) Computed tomography scan pre-initial transcatheter aortic valve (TAV) implantation showing bicuspid anatomy. (**B**) Implantation of the first transcatheter heart valve (too high) with a significant aortic regurgitation. (**C**) Final result of TAV in series. (**D-F**) Computed tomography of pre TAV-in-TAV-in TAV. (**G**) Image before Edwards SAPIEN-3 Ultra (Edwards Lifesciences, Irvine, CA) valve deployment at node 4. (**H**) Postdilatation of the transcatheter heart valve. LCA, left coronary artery; RCA, right coronary artery.

During the current admission, transthoracic echocardiography showed bioprosthetic valve dysfunction with severe valvular AR. Due to the favourable anatomy (high coronary ostia: 17 mm; valve-to-sino-tubular junction ≥ 2 mm; Fig. 1, D-F) and the excellent general condition of the patient, who lives alone at home, we planned an elective TAV-in-TAV-in-TAV procedure for the week after.

However, the patient’s condition rapidly worsened. She developed multiple organ failure (Fig. 2) and acute respiratory distress. After discussion with the patient’s family, we decided to perform a stat redo-TAVI in valves-in-series using a 26-mm SAPIEN-3 Ultra (Edwards Lifesciences, Irvine, CA) without contrast media.

**Figure 2:**
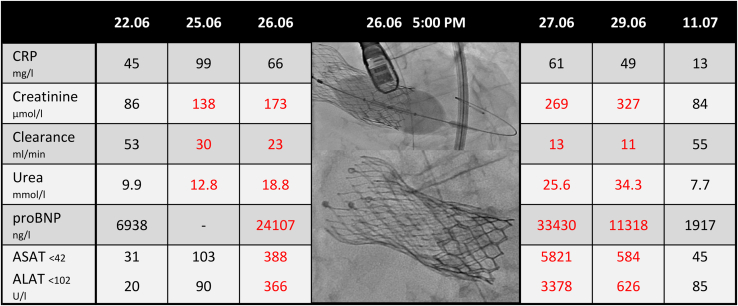
Laboratory tests before and after transcatheter aortic valve (TAV)-in-TAV-in-TAV was performed. Shown are the transcatheter heart valve postdilatation and the final result. ALAT, alanine aminotransferase; ASAT, aspartate aminotransferase; CRP, C-reactive protein; proBNP, N-terminal pro-B type natriuretic peptide.

Before the procedure, the patient was intubated due to severe respiratory distress. To ensure that we crossed the centre of the degenerated THVs without going inside one of the cells of the THV’s high frame, we tried to cross the THV with the pigtail without wire. However, as in the first procedure, crossing the THV was difficult, and we eventually needed a regular wire. Once inside the left ventricle, we tried to mobilize the pigtail from side to side to confirm that it was free (Video 1E , view video online). Subsequently, to eliminate any remaining doubt about the wire trajectory, we advanced a 20-mm Nucleus balloon (NuMED, NY) through the previous THVs on the Safari wire (Boston Scientific, MA). The SAPIEN-3 Ultra valve was advanced and successfully implanted at node 4 with zero contrast media (Fig. 1, G and H; Video 1 , F and G, view video online).

The patient was extubated and admitted to an intermediate care unit where she received a dobutamine infusion and noninvasive ventilation for 48 hours.

The evolution was slowly favourable, with correction of the laboratory tests (Fig. 2) and symptom improvement (mean gradient: 8 mm Hg, with no AR). She needed the implantation of a permanent pacemaker for a complete atrioventricular block on day 2. She was discharged after 7 days to a rehabilitation centre, where she stayed for 2 weeks. At the 6-month assessment, she was living autonomously at home.

In conclusion, emergent redo-TAVI was transformative, considering the impressive clinical evolution despite multiple organ failure in a 97-year-old woman. Redo-TAVI, as well as the TAV-in-TAV-in-TAV procedure, has become an alternative therapy for bioprosthetic valve dysfunction, particularly for patients at high surgical risk when the anatomy is suitable.^1^ Finally, when performing a TAVI on a patient, even, as old as 91 years, the strategy should take into account the potential need for a future redo-TAVI.^2^

**Novel Teaching Points**

• TAV-in TAV-in-TAV is feasible when the anatomy is large enough.
• redo-TAVR without any contrast media is feasible
• The evolution was impressive despite the advanced heart failure with multiple organ failure
• Redo-TAVR has emerged as an alternative therapy for valve dysfunction, particularly for patients at high surgical risk
• When performing a TAVI, the potential need for a future redo-TAVI should be considered (lifetime management)

## Supplementary Material

Video 1 part A: Final valve deployment (29-Evolut Pro) part B: Final result with a valve that popped up during the final release causing a significant AR (AR: aortic regurgitation) part C: Final valve deployment of a second 29-Evolut Pro THV (THV in series) part D: Final result with no residual AR after 29-Evolut Pro THVs in series part E: Check of the free movement of the pigtail catheter inside the valve frame before TAV-in-TAV-in-TAV part F : TAV-in-TAV-in-TAV :Crossing of the prior Evolut-in-series with the 26 mm SAPIEN 3 Ultra Resilia part G: TAV-in-TAV-in-TAV ; Inflation of the 26-mm Sapien 3 Ultra Resilia
